# Limited usefulness of serum carcinoembryonic antigen and carbohydrate antigen 19-9 levels for gastrointestinal and whole-body cancer screening

**DOI:** 10.1038/s41598-020-75319-8

**Published:** 2020-10-23

**Authors:** Masau Sekiguchi, Takahisa Matsuda

**Affiliations:** 1 Cancer Screening Center, National Cancer Center Hospital, 5-1-1 Tsukiji, Chuo-ku, Tokyo, 104-0045 Japan; 2 Division of Screening Technology, Center for Public Health Sciences, National Cancer Center, Tokyo, Japan; 3 Endoscopy Division, National Cancer Center Hospital, Tokyo, Japan

**Keywords:** Cancer screening, Tumour biomarkers

## Abstract

The diagnostic performance of serum carcinoembryonic antigen (CEA) and carbohydrate antigen (CA) 19-9 levels for multiple-organ cancer screening has not been fully elucidated. However, they are widely used for real-world opportunistic screening of multiple-organ cancers. This study aimed to examine the diagnostic performance of these serum markers in multiple-organ cancer screening. Data from asymptomatic individuals subjected to opportunistic cancer screening were analyzed. The diagnostic performance of CEA and CA 19-9 was assessed for (A) upper/lower gastrointestinal cancers and (B) whole-body cancers (including both gastrointestinal and other organ cancers) using the results of upper/lower gastrointestinal endoscopy and whole-body imaging as reference. Data from 12,349 and 7616 screened individuals were used to assess the diagnostic performance of CEA and CA 19-9 for (A) and (B), respectively. For (A), the sensitivity and positive predictive value (PPV) of CEA (cut-off: 5 ng/mL) were 7.8% and 3.7%, respectively; those of CA19-9 (cut-off: 37 U/mL) were 7.4% and 2.7%, respectively. For (B), the sensitivity and PPV of CEA were 6.6% and 4.1%, respectively, and those of CA19-9 were 10.8% and 5.8%, respectively. Considering even multiple cancers, the sensitivity and PPV of CEA and CA 19-9 were low, thus confirming their limited usefulness in multiple-organ cancer screening.

## Introduction

Cancer is the leading cause of death worldwide, and much effort is being put into cancer screening^1^. Noninvasive screening tests that can detect multi-organ cancers simultaneously are ideal; serum tumor markers, which can be easily analyzed via blood sampling, have been expected to be useful for multi-organ cancer screening. However, almost no serum tumor markers are currently recommended for cancer screening^2–6^. Serum carcinoembryonic antigen (CEA) and carbohydrate antigen (CA) 19-9 are the most common tumor markers targeting multiple cancers, including colorectal cancer, gastric cancer, pancreaticobiliary cancer, lung cancer, and breast cancer; they are used in cancer care as prognostic markers and markers for the monitoring of response to therapy and recurrence^2–6^. However, both markers are not recommended by any guidelines for cancer screening, due to their low sensitivity for single-cancer detection at an early stage^2–6^.

Nevertheless, till date, the tumor markers CEA and CA19-9 are widely used for real-world opportunistic cancer screening in several countries including Japan^7–10^. One of the main reasons for their use may be the ambiguous expectation for their usefulness, since they theoretically allow multiple-organ cancer screening. Of note, as mentioned above, the sensitivity of these markers is reportedly low considering each single-organ cancer; however, their diagnostic potential to simultaneously screen multi-organ cancers has not been fully assessed^2–11^. In this context, the utility of these markers for cancer screening may have been underestimated: the assessment of the diagnostic performance for a single-organ cancer does not allow the extrapolation for the multi-organ cancers’ context. Reliable data on the diagnostic performance of these tumor markers as modalities for whole-body cancer screening are therefore warranted, in order to understand whether these markers are useful for cancer screening.

Hence, this study aimed to elucidate the diagnostic performance of serum CEA and CA19-9 for multi-organ cancer screening via the analysis of data obtained from a large number of asymptomatic screened individuals. Their diagnostic abilities for gastrointestinal (GI) cancers, which were reportedly their major target cancers, and whole-body cancers (including not only GI cancers but also cancers of organs other than the GI tract) were assessed by analyzing the data of the screened individuals who underwent tumor marker measurements, esophagogastroduodenoscopy (EGD), total colonoscopy (CS), computed tomography (CT) colonography (CTC), and those who further underwent whole-body imaging tests such as 18-fluoro-2-deoxyglucose positron emission tomography (FDG-PET)^2–11^.

## Methods

### Study design and population

The present study was performed using data retrieved from a database of asymptomatic individuals who underwent cancer screening at the Cancer Screening Center of the National Cancer Center (NCC), Tokyo. Since the foundation of the Cancer Screening Center in February 2004, opportunistic screening for whole-body cancers using multiple modalities has been provided for asymptomatic individuals and the screening results have been prospectively accumulated in the database^12–15^.

Two analyses (1 and 2) were performed. The diagnostic performance of the tumor markers for GI cancer screening (analysis 1) and whole-body cancer screening (analysis 2) was assessed. Among the individuals who underwent cancer screening at the Cancer Screening Center for the first time between February 2004 and December 2018, the data of those who underwent both upper GI (EGD) and lower GI (CS or CTC) examinations, in addition to CEA and CA19-9 serum levels’ measurements were used for analysis 1. In analysis 2, the data of those who further underwent the whole-body FDG-PET test (combined with CT), in addition to EGD, CS/CTC, and CEA/CA19-9 levels’ measurements were used. Principally, they also underwent chest CT, abdominal ultrasonography, and sputum cytology; females among the screened individuals further underwent examinations for breast cancer (mammary ultrasonography, mammography, and palpation) and transvaginal examinations^15^. The data of screened individuals who had a previous history of cancer treatment, refused to participate in the study, and/or were subjected to incomplete examinations were excluded.

### Measurement of tumor marker levels and cancer screening procedures

Cancer screening procedures for each individual were conducted for two consecutive days^12–16^. Blood samples for the determination of tumor marker levels were collected on the first day, and EGD and CS or CTC were performed on the second day, after fasting and bowel preparation. Bowel preparation was performed according to an appropriate method based on the procedures (CS or CTC) as described previously^12–14,16,17^.

For individuals who underwent both EGD and CS, these two endoscopic procedures were performed continuously on the same day. During the endoscopic procedures, an antiperistaltic (10 mg scopolamine butylbromide or 0.5 mg glucagon) was injected intravenously, except when it was contraindicated^12–14,18^. Based on each examinee’s request, a sedative agent (17.5–35 mg pethidine hydrochloride or 2–10 mg midazolam) was also used. Before EGD, 100 mL of solution containing 1 g of Pronase and 1 g of sodium bicarbonate was administered to the examinees to remove mucus and bubbles from the mucosa of the upper GI tract^18^. For EGD and CS observations, image-enhanced endoscopy, including chromoendoscopy, was routinely performed. In addition, magnification was used for high-quality diagnosis during CS^12–14^. All lesions that appeared potentially malignant were examined histopathologically.

For individuals undergoing whole-body imaging examinations, FDG-PET, chest CT, abdominal ultrasonography, sputum cytology, mammary palpation and ultrasonography, mammography, and transvaginal examinations were performed on the first day^12,15,18–20^. The FDG-PET examination was conducted according to the Japanese FDG-PET guidelines published by the Japanese Society of Nuclear Medicine (https://jsnm.org/useful/guidelines). A significantly higher round or oval focal accumulation of FDG than the background level was considered a positive finding^12,20^. When a positive finding was obtained, an examinee was referred to a hospital for further work-up examinations.

All the screening procedures were performed by experienced specialists, also responsible for their interpretation, and the consequent diagnoses^12–20^.

The cut-off values for CEA and CA19-9 positivity were set at 5 ng/mL and 37 U/mL, respectively, according to previous studies; values higher than cut-off ones were considered positive in the tumor marker tests^2–11^. EGD, CS/CTC, as well as the remaining tests were performed in a blinded manner to the results of the tumor markers.

### Assessment of the diagnostic performance of serum CEA and CA19-9 levels in the screening of GI cancer (analysis 1) and whole-body cancer (analysis 2)

In analysis 1, the diagnostic performance of the tumor markers was assessed based on the results of the marker measurement and the information of upper and lower GI cancers detected by EGD and CS/CTC. Because EGD and CS/CTC are established as useful modalities to accurately detect upper and lower GI cancers, respectively, the results of these modalities were used^21–25^.

All upper and lower GI cancers were confirmed pathologically. For upper GI cancers, considering the reported target of the tumor markers, their diagnostic performance for gastric cancer and other adenocarcinomas (esophageal and duodenal cancers), excluding esophageal squamous cell carcinoma, were evaluated^2–11^. For lower GI cancers, their diagnostic performance for colorectal cancer was evaluated. After obtaining their pathological diagnosis following treatment, the diagnostic accuracy of the tumor markers for GI cancers according to the depth of invasion (all including intramucosal cancer/cancer with invasion to submucosa or deeper) was also assessed.

In analysis 2, the results of the tumor marker measurements and those of cancers detected by EGD, CS/CTC, FDG-PET, and other previously mentioned screening tests were compared. GI, pancreaticobiliary, lung, breast, thyroid, gynecological, and bladder cancers were assessed, and their diagnoses were pathologically confirmed in the work-up examination and treatment after screening.

### Association of participant characteristics and presence of cancer with tumor markers’ positivity

The relationships between tumor markers’ positivity (CEA > 5 ng/mL, CA19-9 > 37 U/mL) and the characteristics of the screened individuals [age, sex, body mass index (BMI), and smoking and alcohol drinking status] were assessed using the data obtained from analysis 2. They were evaluated after adjusting for the effects of other characteristics and the presence of cancers.

The association between the presence of GI/ whole-body cancers and tumor markers’ positivity was also evaluated after adjusting for the effect of the participants’ characteristics.

### Statistical analysis

For assessing the diagnostic performance of the tumor markers, sensitivity, specificity, positive predictive value (PPV), and negative predictive value (NPV) were calculated with 95% confidence intervals (CIs). The cut-off values for the tumor markers used included the previously mentioned values used in the daily practice (CEA: 5 ng/mL and CA19-9: 37 U/mL) and additionally twice these values (CEA: 10 ng/mL and CA19-9: 74 U/mL)^2–11^. The diagnostic performance of the tumor markers for cancer diagnosis was also examined using receiver operating characteristic curves and c-statistics.

The association of the participant characteristics and presence of cancers with the tumor markers’ positivity was evaluated using the chi-square test. Multivariate logistic regression was also performed to estimate the adjusted odds ratio (OR) of each factor for tumor markers’ positivity.

All statistical analyses were performed using the SPSS software (version 26.0; IBM Corp., Armonk, NY, USA), and EZR, version 1.50 (Saitama Medical Center, Jichi Medical University, Japan), which is a graphical user interface for R (The R Foundation for Statistical Computing, Vienna, Austria)^26^.

### Ethics approval and consent to participate

This study was approved by the Ethics Committee for Clinical Research of the National Cancer Center Hospital in Tokyo, Japan (2016-166). We conducted this study in accordance with the Declaration of Helsinki. Informed consent was obtained from each participant.

## Results

### Characteristics of the study participants

**Figure 1:**
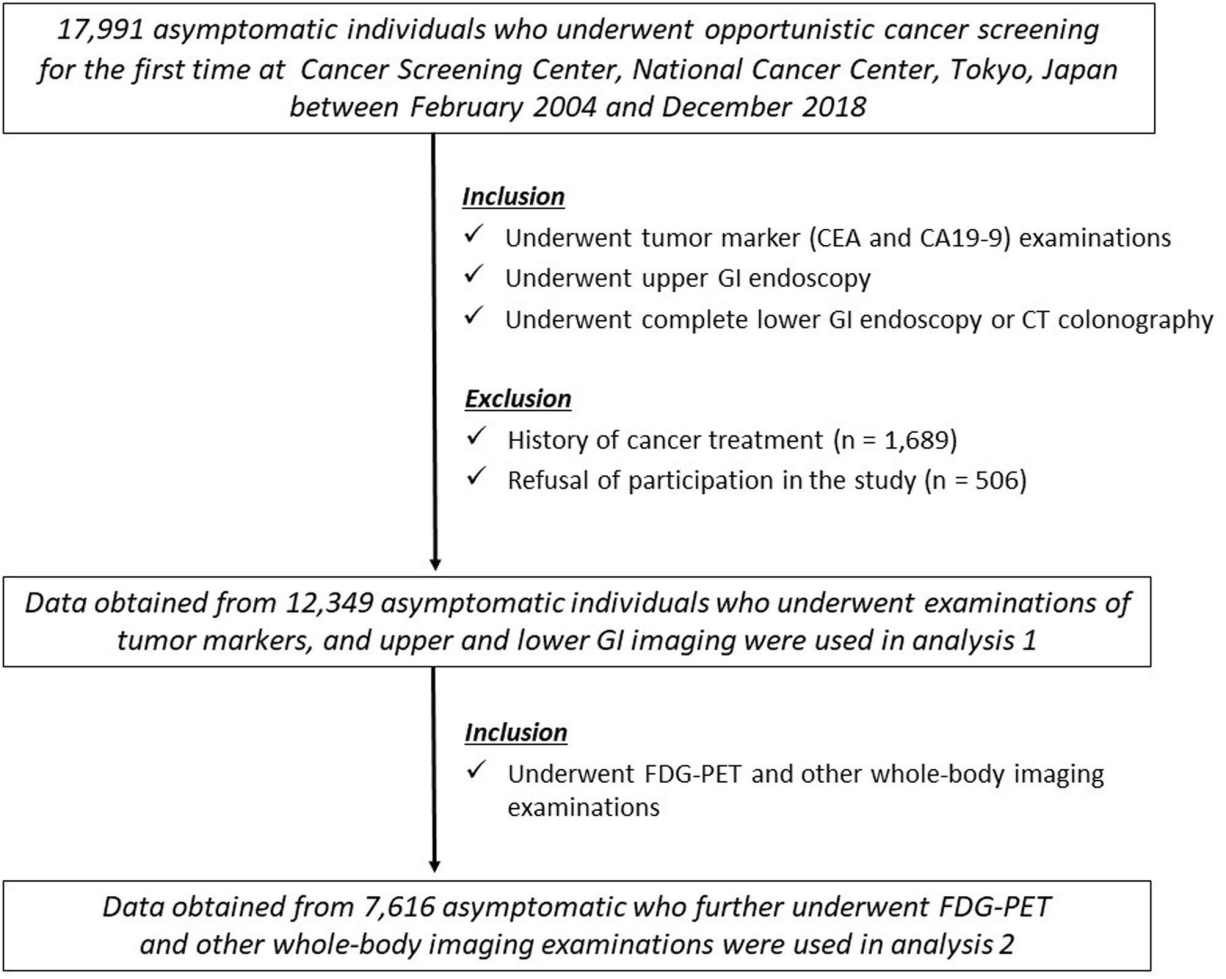
Flowchart of selection of the data used in this study. *CA19-9* carbohydrate antigen 19-9, *CEA* carcinoembryonic antigen, *CT* computed tomography, *FDG-PET* 18-fluoro-2-deoxyglucose positron emission tomography, *GI* gastrointestinal.

The flowchart included as Fig. 1 explains the selection process of the data used in this study. Data collected from 12,349 screened individuals were used to perform analysis 1. Among them, data obtained from 7616 individuals were used for analysis 2. The characteristics and tumor markers’ measurements of the study participants are summarized in Table 1. The proportions of positive CEA (> 5 ng/mL) and CA19-9 (> 37 U/mL) were approximately 4% and 5%, respectively. The numbers and proportions of individuals with cancers are also described in Table 1. In analysis 1, 230 individuals (1.9%) had upper or lower GI cancers (gastric cancer, duodenal cancer, and colorectal cancer). In analysis 2, 213 individuals (2.8%) had at least one of whole-body cancers (GI, pancreatic, lung, breast, thyroid, gynecological, or bladder cancer). No esophageal adenocarcinoma and biliary cancer were detected.

**Table 1:** Characteristics of the study participants.

	All study participants (for analysis 1)	Study participants who underwent whole-body imaging (for analysis 2)
	n = 12,349	n = 7616
**Age, years old,** **median (interquartile range)**	58 (50–64)	59 (52–65)
**Sex, n (%)**		
Male	7894 (63.9)	4919 (64.6)
Female	4455 (36.1)	2697 (35.4)
**BMI, kg/m**^**2**^**, median (interquartile range)**	23.1 (21.2–25.1)	23.3 (21.3–25.3)
**Smoking, n (%)**		
Current smoker	1658 (13.4)	1059 (13.9)
Ex-smoker	4466 (36.1)	2794 (36.7)
Non-smoker	6232 (50.4)	3763 (49.4)
**Alcohol drinking, n (%)**		
Current drinker	9047 (73.3)	5495 (72.2)
Ex-/non-drinker	3302 (26.7)	2121 (27.8)
**CEA value, ng/mL, median (interquartile range)**	1.8 (1.2–2.8)	1.8 (1.2–2.8)
**CEA value range, n (%)**		
> 10 ng/mL	51 (0.4)	38 (0.5)
> 5 and ≤ 10 ng/mL	436 (3.5)	306 (4.0)
≤ 5 ng/mL	11,862 (96.1)	7272 (95.5)
**CA19-9 value, U/mL, median (interquartile range)**	9 (6–16)	9 (6–17)
**CA19-9 value range, n (%)**		
> 74 U/mL	82 (0.7)	55 (0.7)
> 37 and ≤ 74 U/mL	548 (4.4)	343 (4.5)
≤ 37 U/mL	11,719 (94.9)	7,228 (94.9)
**Number of individuals with gastric cancer, n (%)**	88 (0.7)	63 (0.8)
T1a*/others	60 (0.5)/28 (0.2)	41 (0.5)/22 (0.3)
**Number of individuals with colorectal cancer, n (%)**	142 (1.1)	85 (1.1)
Tis*/others	107 (0.9)/35 (0.3)	64 (0.8)/21 (0.3)
**Number of individuals with duodenal cancer, n (%)**	1 (0.0)	1 (0.0)
Tis*/others	1 (0.0)/0 (0.0)	1 (0.0)/0 (0.0)
**Number of individuals with pancreatic cancer, n (%)**	–	4 (0.1)
Tis*/others	–	0 (0.0)/4 (0.1)
**Number of individuals with lung cancer, n (%)**	–	18 (0.2)
Tis*/others	–	1 (0.0)/17 (0.2)
**Number of individuals with breast cancer, n (%)**	–	27 (0.4)
Tis*/others	–	11 (0.1)/16 (0.2)
**Number of individuals with other cancers, n (%)**	–	18 (0.2)
Thyroid cancer	–	7 (0.1)
Gynecological cancer	–	6 (0.1)
Bladder cancer	–	5 (0.1)

*T classification is based on the TNM classification of malignant tumors by Union for International Cancer Control, 8th ed.

*CA19-9* carbohydrate antigen 19-9, *CEA* carcinoembryonic antigen.

### Diagnostic performance of the tumor markers for GI cancer screening (analysis 1)

The results on the diagnostic performance of the tumor markers for GI cancer screening are summarized in Table 2. The c-statistics of the markers for the cancers were low, ranging between 0.51 and 0.63. Even with GI cancers, the median marker values were lower than the cut-off values for positivity. The sensitivity and PPV of the tumor markers for GI cancers were also very low; those of CEA (cut-off: 5 ng/mL) for upper/lower GI cancers were 7.8% and 3.7%, respectively, and those of CA19-9 (cut-off: 37 U/mL) were 7.4% and 2.7%, respectively. Even when the high cut-off values were adopted (CEA: 10 ng/mL, and CA19-9: 74 U/mL), the PPVs were still low: 11.8% and 4.9%, for CEA and CA19-9, respectively.

**Table 2:** Diagnostic performance of the tumor markers in gastrointestinal cancer screening (analysis 1).

	Gastric cancer		Colorectal cancer		Upper/lower GI cancer		No upper or lower GI cancer
	Cancer with invasion to submucosa or deeper	All	Cancer with invasion to submucosa or deeper	All	Cancer with invasion to submucosa or deeper	All	
	n = 28	n = 88	n = 35	n = 142	n = 63	n = 230	n = 12,119
**CEA**							
**Number of individuals according to the CEA range, ng/mL**							
> 10	2	2	2	4	4	6	45
> 5 and ≤ 10	0	5	2	7	2	12	424
≤ 5	26	81	31	131	57	212	11,650
**CEA value, ng/mL**, median (IQR)	2.0 (1.5–3.2)	2.1 (1.5–3.4)	2.3 (1.6–3.9)	2.1 (1.5–3.3)	2.1 (1.6–3.6)	2.1 (1.5–3.4)	1.8 (1.2–2.7)
**C-statistic of CEA **(95% CI)	0.57 (0.46–0.67)	0.60 (0.54–0.66)	0.63 (0.54–0.72)	0.58 (0.53–0.63)	0.60 (0.53–0.67)	0.59 (0.55–0.63)	–
**Diagnostic accuracy of CEA (cut-off 5 ng/mL)**							
Sensitivity	7.1%	8.0%	11.4%	7.7%	9.5%	**7.8%**	–
Specificity	96.1%	96.0%	96.1%	96.1%	96.1%	**96.1%**	–
PPV	0.4%	1.4%	0.8%	2.3%	1.2%	**3.7%**	–
NPV	99.8%	99.3%	99.7%	98.9%	99.5%	**98.2%**	–
**Diagnostic accuracy of CEA (cut-off 10 ng/mL)**							
Sensitivity	7.1%	2.3%	5.7%	2.8%	6.3%	**2.6%**	–
Specificity	99.6%	99.6%	99.6%	99.6%	99.6%	**99.6%**	–
PPV	3.9%	3.9%	3.9%	7.8%	7.8%	**11.8%**	–
NPV	99.8%	99.3%	99.7%	98.9%	99.5%	**98.2%**	–
**CA 19-9**							
**Number of individuals according to the CA19-9 range, U/mL**							
> 74	2	3	1	1	3	4	78
> 37 and ≤ 74	3	6	4	7	7	13	535
≤ 37	23	79	30	134	53	213	11,506
**CA19-9 value, U/mL**, median (IQR)	13.5 (7.3–28.5)	11.0 (7.0–18.8)	8.0 (4.0–14.0)	8.5 (5.0–15.0)	9.0 (5.0–18.0)	10.0 (6.0–18.0)	9.0 (6.0–16.0)
**C-statistic of CA19-9** (95%CI)	0.60 (0.49–0.72)	0.56 (0.51–0.62)	0.55 (0.45–0.66)	0.52 (0.48–0.57)	0.52 (0.44–0.60)	0.51 (0.48–0.55)	–
**Diagnostic accuracy of CA19-9 (cut-off 37 U/mL)**							
Sensitivity	17.9%	10.2%	14.3%	5.6%	15.9%	**7.4%**	–
Specificity	94.9%	94.9%	94.9%	94.9%	95.0%	**94.9%**	–
PPV	0.8%	1.4%	0.8%	1.3%	1.6%	**2.7%**	–
NPV	99.8%	99.3%	99.7%	98.9%	99.5%	**98.2%**	–
**Diagnostic accuracy of CA19-9 (cut-off 74 U/mL)**							
Sensitivity	7.1%	3.4%	5.7%	1.4%	4.8%	**1.7%**	–
Specificity	99.4%	99.3%	99.3%	99.3%	99.4%	**99.4%**	–
PPV	2.4%	3.7%	1.2%	1.2%	3.7%	**4.9%**	–
NPV	99.8%	99.3%	99.7%	98.9%	99.5%	**98.2%**	–

*CA19-9* carbohydrate antigen 19-9, *CEA* carcinoembryonic antigen, *NPV* negative predictive value, *PPV* positive predictive value.

### Diagnostic performance of the tumor markers for whole-body cancer screening (analysis 2)

Table 3 summarizes the results of the diagnostic performance of the tumor markers for whole-body cancer screening. All c-statistics of the tumor markers for the examined cancers were lower than 0.7, except the value of CA19-9 for pancreatic cancer (0.85, n = 4). The tumor markers had very low sensitivity and PPV for whole-body cancers; those of CEA (cut-off: 5 ng/mL) were 6.6% and 4.1%, respectively, and those of CA19-9 (cut-off: 37 U/mL) were 10.8% and 5.8%, respectively. Even with the high cut-off values of the tumor markers (CEA: 10 ng/mL, CA19-9: 74 U/mL), PPV were still low for whole-body cancers: the PPV of CEA and CA19-9 were 13.2% and 14.5%, respectively.

**Table 3:** Diagnostic performance of the tumor markers in whole-body cancer screening (analysis 2).

	Whole-body cancer					No cancer
	GI cancer	Pancreatic cancer	Lung cancer	Breast cancer	All	
	n = 148	n = 4	n = 18	n = 27	n = 213	n = 7,403
**CEA**						
**Number of individuals according to the CEA range, ng/mL**						
> 10	3	0	2	0	5	33
> 5 and ≤ 10	7	0	1	0	9	297
≤ 5	138	4	15	27	199	7,073
**CEA value**, **ng/mL**, median (IQR)	2.3 (1.4–3.4)	2.4 (1.9–3.8)	2.6 (2.0–3.5)	1.8 (1.1–2.7)	2.1 (1.5–3.3)	1.8 (1.2–2.8)
**C-statistic of CEA**	0.58 (0.54–0.63)	0.68 (0.50–0.85)	0.69 (0.59–0.79)	0.47 (0.35–0.58)	0.57 (0.53–0.61)	–
**Diagnostic accuracy of CEA (cut-off 5 ng/mL)**						
Sensitivity	6.8%	0.0%	16.7%	0.0%	**6.6%**	–
Specificity	95.5%	95.5%	95.5%	95.5%	**95.5%**	–
PPV	2.9%	0.0%	0.9%	0.0%	**4.1%**	–
NPV	98.1%	99.9%	99.8%	99.6%	**97.3%**	–
**Diagnostic accuracy of CEA (cut-off 10 ng/mL)**						
Sensitivity	2.0%	0.0%	11.1%	0.0%	**2.3%**	–
Specificity	99.5%	99.5%	99.5%	99.5%	**99.6%**	–
PPV	7.9%	0.0%	5.3%	0.0%	**13.2%**	–
NPV	98.1%	100.0%	99.8%	99.6%	**97.3%**	–
**CA 19-9**						
**Number of individuals according to the CA19-9 range, U/mL**						
> 74	4	0	0	1	8	47
> 37 and ≤ 74	7	1	2	4	15	328
≤ 37	137	3	16	22	190	7,028
**CA19-9 value**, **U/mL** median (IQR)	10.0 (6.0–16.8)	27.0 (15.8–39.8)	14.5 (9.5–22.0)	12.0 (8.0–26.0)	11.0 (6.0–20.0)	9.0 (6.0–16.0)
**C-statistic of CA19-9**	0.51 (0.46–0.55)	0.85 (0.72–0.98)	0.68 (0.58–0.78)	0.65 (0.55–0.75)	0.56 (0.52–0.59)	–
**Diagnostic accuracy of CA19-9 (cut-off 37 U/mL)**						
Sensitivity	7.4%	25.0%	11.1%	18.5%	**10.8%**	–
Specificity	94.8%	94.8%	94.8%	94.8%	**94.9%**	–
PPV	2.8%	0.3%	0.5%	1.3%	**5.8%**	–
NPV	98.1%	100.0%	99.8%	99.7%	**97.4%**	–
**Diagnostic accuracy of CA19-9 (cut-off 74 U/mL)**						
Sensitivity	2.7%	0.0%	0.0%	3.7%	**3.8%**	–
Specificity	99.3%	99.3%	99.3%	99.3%	**99.4%**	–
PPV	7.3%	0.0%	0.0%	1.8%	**14.5%**	–
NPV	98.1%	100.0%	99.8%	99.7%	**97.3%**	–

*CA19-9* carbohydrate antigen 19-9, *CEA* carcinoembryonic antigen, *GI* gastrointestinal cancer, *NPV* negative predictive value, *PPV* positive predictive value.

### Combined use of the tumor markers for GI and whole-body cancer screening (analyses 1 and 2)

The numbers and proportions of GI and whole-body cancers, as per the results of the combined use of CEA and CA19-9 for cancer screening are given in Table 4. Even when both the markers were positive (CEA > 5 ng/mL and CA19-9 > 37 U/mL), the PPV for GI and whole-body cancers were very low (3.0% and 4.4%, respectively). However, when the higher CEA and CA19-9 cut off levels were considered (> 10 ng/mL, and > 74 U/mL, respectively), the PPVs were high; of note, in this context, the number of cases was low.

**Table 4:** Combined use of the tumor markers in gastrointestinal and whole-body cancer screening.

	Analysis 1			Analysis 2					
	Total number of individuals	Individuals with GI cancer (with invasion to submucosa or deeper)	Individuals with GI cancer (all)	Total number of individuals	Individuals with GI cancer (all)	Individuals with pancreatic cancer	Individuals with lung cancer	Individuals with breast cancer	Individuals with all cancer
CEA > 10 ng/mL and CA19-9 > 74 U/mL	3	2 (**66.7%**)	2 (**66.7%**)	2	2 (**100.0%**)	0 (0.0%)	0 (0.0%)	0 (0.0%)	2 (**100.0%**)
CEA > 5 ng/mL and CA19-9 > 37 U/mL	67	2 (**3.0%**)	2 (**3.0%**)	45	2 (**4.4%**)	0 (0.0%)	0 (0.0%)	0 (0.0%)	2 (**4.4%**)
CEA ≤ 5 ng/mL and CA19-9 ≤ 37 U/mL	11,299	49 (**0.4%**)	197 (**1.7%**)	6919	129 (**1.9%**)	3 (0.0%)	13 (0.2%)	22 (0.3%)	178 (**2.6%**)

*CA19-9* carbohydrate antigen 19-9, *CEA* carcinoembryonic antigen, *GI* gastrointestinal.

### Association of participant characteristics and presence of cancer with tumor markers’ positivity

The association of participant characteristics and presence of cancer with tumor markers’ positivity is summarized in Table 5.

**Table 5:** Association of participant characteristics and presence of cancer with tumor markers’ positivity.

	CEA			CA19-9		
	Proportion of individuals with CEA > 5	Adjusted Odds ratio	Adjusted P value	Proportion of individuals with CA19-9 > 37	Adjusted Odds ratio	Adjusted P value
**Age, years**			< 0.001			0.017
≥ 60	5.9%	2.2 (1.7–2.7)		6.0%	1.3 (1.0–1.6)	
< 60	3.3%	1		4.5%	1	
**Sex**			0.211			< 0.001
Male	5.2%	1.2 (0.9–1.6)		3.9%	1	
Female	3.2%	1		7.7%	1.8 (1.4–2.3)	
**BMI, kg/m**^**2**^			0.141			0.002
≥ 25	4.2%	0.8 (0.6–1.1)		3.5%	1	
< 25	4.7%	1		5.9%	1.5 (1.2–2.0)	
**Smoking**						
Current smoker	12.5%	5.4 (4.0–7.2)	< 0.001	3.9%	0.9 (0.6–1.3)	0.507
Ex-smoker	3.9%	1.3 (1.0–1.8)	0.100	4.3%	1.0 (0.8–1.3)	0.919
Non-smoker	2.8%	1		6.3%	1	
**Alcohol drinking**			0.851			0.007
Current drinker	4.7%	1.0 (0.7–1.3)		4.4%	1	
Ex-/non-drinker	4.0%	1		7.3%	1.4 (1.1–1.7)	
**GI cancer**			0.737			0.188
Present	6.8%	1.1 (0.6–2.2)		7.4%	1.5 (0.8–2.8)	
Absent	4.5%	1		5.2%	1	
**All cancer**			0.475			0.001
Present	6.6%	1.2 (0.7–2.2)		10.8%	2.2 (1.4–3.4)	
Absent	4.5%	1		5.1%	1	

*CA19-9* carbohydrate antigen 19-9, *CEA* carcinoembryonic antigen, *GI* gastrointestinal.

Age (> 60 years) and “currently smoking” status were identified as independent risk factors for CEA positivity; particularly the latter was a strong risk factor with an adjusted OR of 5.4 (95% CI 4.0–7.2). However, the presence of at least one GI cancer and at least one cancer in the whole body were not significantly associated with CEA positivity after adjusting for other factors.

Age (> 60 years), sex (female), BMI (< 25 kg/m^2^), and ex-/non-alcohol drinking habits were significantly associated with CA 19-9 positivity. Moreover, the presence of at least one cancer in the whole body was associated with CA19-9 positivity even after adjusting for other factors, with an adjusted OR of 2.2 (95% CI 1.4–3.4).

## Discussion

The present study explored the diagnostic performance of the tumor markers CEA and CA19-9 in GI and whole-body cancer screening, and clearly demonstrated their very low sensitivities and PPV even when targeting multiple GI and whole-body cancers instead of single-organ cancers. The use of these tumor markers is not recommended for the screening of single cancers such as colorectal cancer, pancreatic cancer, etc. in any guidelines^2–6^. However, they are widely used in real-world opportunistic cancer screening in several countries, with ambiguous expectations for their usefulness as screening modalities for whole-body cancers. Of note, in several countries, including Japan, the work-up examinations following abnormal findings on these tumor markers are conducted under the national health insurance funding. Our findings pertaining to the highly limited usefulness of these markers as multiple-cancer screening modalities indicate the necessity of reconsidering their use in real-world cancer screening.

A limited number of studies have examined the utility of these tumor markers as whole-body cancer screening modalities and suggested their limited usefulness^8–10^. Our study confirmed their limited usefulness based on the assessment of data collected from a large number of asymptomatic screened individuals, including the results of high-quality endoscopic and multiple whole-body examinations, such as EGD, CS, CTC, FDG-PET, chest CT, abdominal ultrasonography and breast imaging that were used as a reference. To the best of our knowledge, this is the first report to confirm the limited usefulness of these tumor markers as multiple-cancer screening modalities based on the results of a variety of reliable diagnostic examinations.

Another strength of this study is the fact that not only the diagnostic performance (sensitivity, specificity, PPV, and NPV) of the tumor markers was evaluated based on standard cut-off values, but also other informative findings were obtained. First, this study clarified that the PPVs of these markers do not increase sufficiently even when more conservative cut-off values are considered (twice the standard values). This emphasizes the difficulty to use these markers as screening modalities regardless of the cut-off values. Second, the discriminatory abilities of these markers for cancers were examined via c-statistics analysis. The c-statistics of these markers for GI and whole-body cancers were low, indicating that their discriminatory abilities are limited. Only the c-statistics of CA19-9 for pancreatic cancer were relatively high (0.85), indicating its potential discriminatory ability for this particular cancer. However, the discriminatory ability of this marker is difficult to be confirmed because of the small number of pancreatic cancer cases (n = 4) studied, and considering that most of the pancreatic cancer cases (3 out of 4) showed normal CA19-9 levels; therefore CA19-9 may not satisfactorily detect pancreatic cancer, as described in the guidelines and in previous studies^2–10^. Third, the diagnostic performance of the combined use of CEA and CA19-9 was also examined. Interestingly, even when both markers were positive, the number of asymptomatic screened individuals diagnosed with any kind of cancer was still very low. On the contrary, some individuals with cancer showed normal levels of both markers. This said [despite the limited number of cases (n = 3)], this study suggests that when both markers show very high values (CEA > 10 ng/mL and CA19-9 > 74 U/mL), the possibility of having cancer may be high.

Furthermore, the baseline characteristics of the screened individuals were assessed for their relationships with tumor markers’ positivity. Consistently with the results of previous studies, smoking was strongly associated with CEA positivity^27,28^. In addition, the relationships between other factors and tumor markers’ positivity were elucidated, as summarized in Table 5. These findings indicate the difficulty of using these markers as screening modalities because of the effect of other baseline characteristics. After adjusting for the effect of other characteristics, no significant relationships were observed between GI and whole-body cancers and CEA, and between GI cancers and CA19-9. However, a significant association was observed between CA19-9 and the presence of whole-body cancers; still, the proportion of individuals showing CA19-9 positivity among those with at least one type of cancer was not high (10.8%). These findings also strengthen the conclusion that these tumor markers are inappropriate to be used as screening modalities for GI and whole-body cancers.

Although CEA and CA19-9, the tumor markers that are most widely used, were found to have a poor diagnostic ability for the screening of GI and whole-body cancers in this study, several other potential markers, including hematopoietic growth factors, enzymes, circulating tumor cells, and genetic markers, are being developed or evaluated^29^. For instance, with respect to enzymes, the activity of alcohol dehydrogenase is reportedly different in patients with cancers such as gastric cancer, colorectal cancer, etc^29–31^. Regarding genetic markers, recently, microRNAs have garnered attention as a potential useful marker for cancer screening^32^. Further evaluation of these potential markers, including an assessment of their diagnostic performance in an asymptomatic screening population, is warranted.

This study has several limitations. First, a single-center database was used, which excluded external validity. Second, although the results of various established examinations were used as a reference, there is a possibility that some types of cancer may have been missed. However, considering that each examination conducted in this study has high detectability of cancers and that all study participants underwent multiple examinations (for instance, lung cancer was examined via chest CT, sputum cytology, and FDG-PET, and pancreatic cancer screened using abdominal US and FDG-PET), at least the possibility of having missed detecting advanced cancers is presumably little^21–25,33,34^. Third, not all characteristics of the screened individuals, including comorbidities, were assessed in this study. There may be other factors that affect tumor markers’ levels, such as benign liver and biliary diseases, that makes the use of these markers for cancer screening even less appealing^2–6^.

In conclusion, the limited usefulness of the tumor markers CEA and CA19-9 in GI and whole-body cancer screening was confirmed in this study. Of note, their use is difficult to consider even for the screening of multiple cancers, and thus, the real-world use of these markers in opportunistic cancer screening should be reconsidered.

## Data Availability

All analyses relevant to the study are included in the article. All data requests should be submitted to the corresponding author for consideration. Access to anonymised data may be granted following review.
